# A novel recanalization technique using inverted guidewire puncture under cholangioscopy for complete biliary stricture after liver transplantation

**DOI:** 10.1055/a-2779-7511

**Published:** 2026-02-03

**Authors:** Qing Yan, Hao Zhou, Yan Zhang, Guoliang Zhao

**Affiliations:** 1 66310 Department of Gastroenterology, The First Affiliated Hospital of Shandong First Medical University and Shandong Provincial Qianfoshan Hospital, Jinan, China; 2 66310 Department of Plastic Surgery, The First Affiliated Hospital of Shandong First Medical University and Shandong Provincial Qianfoshan Hospital, Jinan, China

The overall incidence of biliary strictures ranges from 10 to 37% after liver transplantation 1 . Endoscopic retrograde cholangiopancreatography (ERCP) is a highly effective therapy for biliary complications after liver transplantation, but in some cases, the initial therapy may fail because of the inability of guidewires to pass through the stricture 2 3 4 . We report here a successful endoscopic recanalization of complete biliary obstruction after liver transplantation using inverted guidewire puncture under cholangioscopy.

**Fig. 1:**
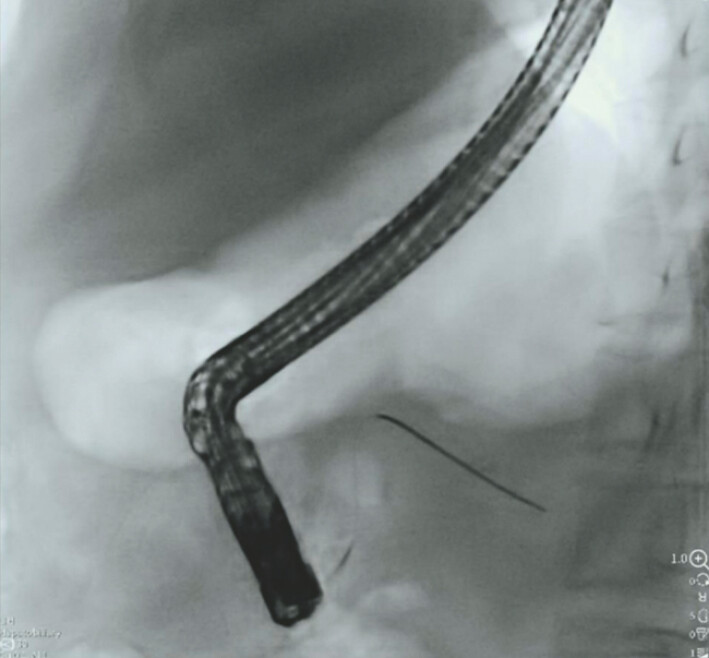
The guidewire had entered the pancreatic duct.

A 50-year-old man, after liver transplantation for the liver malignant tumor 11 months ago, was referred for ERCP for the biliary stricture ( Video 1 ). Selective cannulation of the bile duct was attempted using a sphincterotome over guidewires. ERCP showed that the guidewire had entered the pancreatic duct multiple times ( Fig. 1 ). To prevent pancreatitis, a pancreatic stent was placed endoscopically.

Further attempts were made to puncture the bile duct using the inverted guidewire.Video 1

**Fig. 2:**
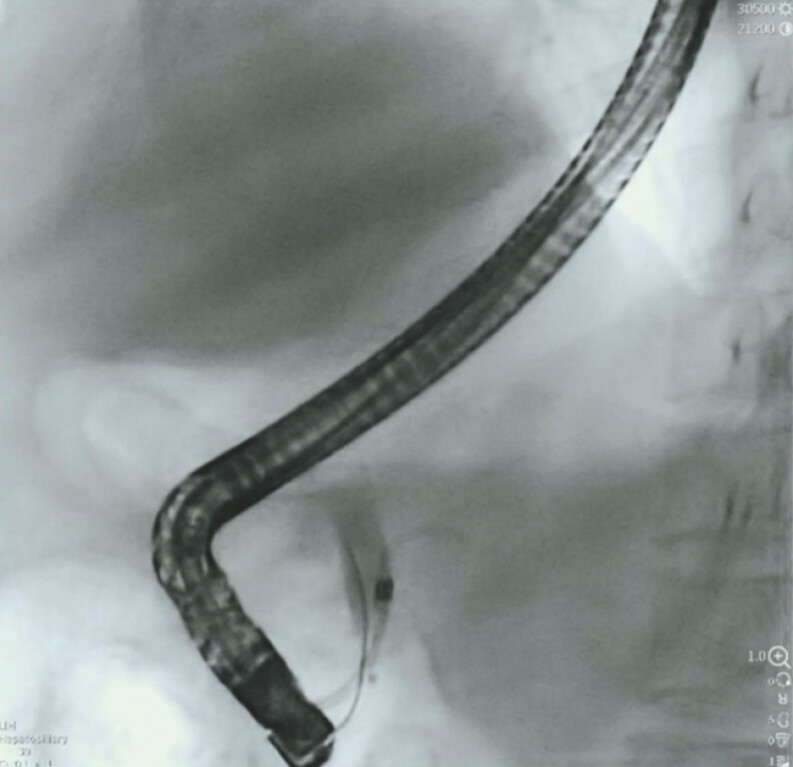
Cholangiography showed the complete obstruction of the bile duct with inability for contrast to pass.

A new guidewire was then successfully inserted into the common bile duct after precut sphincterotomy of the duodenal papilla with a needle-knife. Upon successful cannulation, cholangiography showed the complete obstruction of the bile duct with inability for contrast to pass ( Fig. 2 ).

**Fig. 3:**
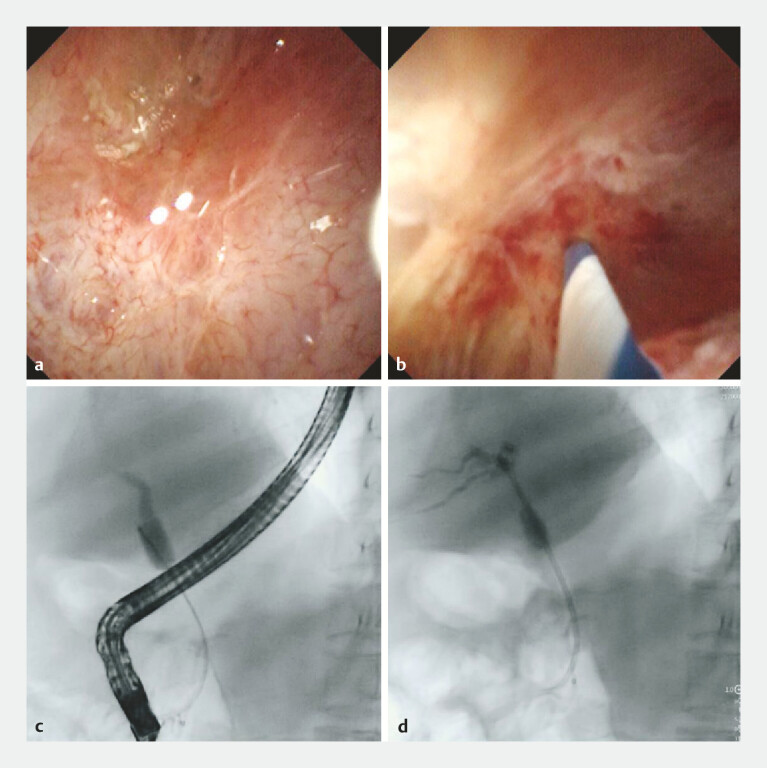
The inverted guidewire was used to puncture under cholangioscopy for the complete biliary stricture. **a** Biliary endoscopy was performed and confirmed complete biliary occlusion. **b** Further attempts were made to puncture the bile duct using the inverted guidewire. **c** The guidewire successfully accessed the intrahepatic bile duct. **d** A plastic biliary stent was placed over the guidewire, with the fluent drainage of bile juice.

Biliary endoscopy was performed and confirmed complete biliary occlusion. Under direct cholangioscopic visualization, guidewire cannulation across the occluded segment was attempted multiple times but unsuccessful. Subsequently, the guidewire was reversed, and further attempts were made using its stiff end to recanalize the bile duct. After several attempts, the stiff end of the guidewire successfully accessed the intrahepatic bile duct. Cholangiography revealed dilation of the intrahepatic biliary system. A plastic biliary stent (7 Fr, 10 cm) was then placed over the guidewire, with the fluent drainage of bile juice ( Fig. 3 ).

This patient showed marked improvement following ERCP without complications such as pancreatitis, bile leak, perforation, and gastrointestinal bleeding. This puncture method offers the following advantages: (1) The penetrating instrument is the stiff end of a guidewire, which requires no special additional devices, thereby reducing the cost. (2) It is performed under direct visualization, allowing the biliary endoscope to adjust the puncture direction and avoid blind maneuvers.

Endoscopy_UCTN_Code_TTT_1AR_2AG
